# Biomineralization of Engineered Spider Silk Protein-Based Composite Materials for Bone Tissue Engineering

**DOI:** 10.3390/ma9070560

**Published:** 2016-07-11

**Authors:** John G. Hardy, Jose Guillermo Torres-Rendon, Aldo Leal-Egaña, Andreas Walther, Helmut Schlaad, Helmut Cölfen, Thomas R. Scheibel

**Affiliations:** 1 Lehrstuhl Biomaterialien, Universität Bayreuth, Universitätsstraße 30, Bayreuth 95447, Germany; johnhardyuk@gmail.com (J.G.H.); aldoleal@yahoo.com (A.L.-E.); 2 DWI Leibniz Institute for Interactive Materials, Forckenbeckstr. 50, Aachen 52056, Germany; torres@dwi.rwth-aachen.de (J.G.T.-R.); walther@dwi.rwth-aachen.de (A.W.); 3 Institute of Chemistry, University of Potsdam, Karl-Liebknecht-Str. 24-25, Potsdam 14476, Germany; schlaad@uni-potsdam.de; 4 Physical Chemistry, Department of Chemistry, University of Konstanz, Universitätsstr. 10, Konstanz D-78457, Germany

**Keywords:** spider silk, recombinant protein, biodegradable polymers, biomaterials, biomineralization, bone tissue engineering

## Abstract

Materials based on biodegradable polyesters, such as poly(butylene terephthalate) (PBT) or poly(butylene terephthalate-*co*-poly(alkylene glycol) terephthalate) (PBTAT), have potential application as pro-regenerative scaffolds for bone tissue engineering. Herein, the preparation of films composed of PBT or PBTAT and an engineered spider silk protein, (eADF4(C16)), that displays multiple carboxylic acid moieties capable of binding calcium ions and facilitating their biomineralization with calcium carbonate or calcium phosphate is reported. Human mesenchymal stem cells cultured on films mineralized with calcium phosphate show enhanced levels of alkaline phosphatase activity suggesting that such composites have potential use for bone tissue engineering.

## 1. Introduction

Bones are composed of mixtures of inorganic material, predominantly calcium phosphate in the form of carbonated hydroxyapatite, and organic material, predominantly collagen, and many different materials and manufacturing methodologies are used in the development of bone tissue scaffolds [1]. While non-biodegradable materials (e.g., metals, polyethylene and polyetheretherketone [2,3]) are commonly used to manufacture components for certain applications in bone tissue, for instance hip replacements, there are issues with these materials, such as inflammation, metal sensitivity and toxicity, and solutions to these issues are the subject of ongoing research [2,3]. Biodegradable materials are of particular interest because their eventual resorption allows them to be remodeled in vivo, and biodegradable polymer-based materials and composites based thereon are popular avenues of research [4,5,6,7,8,9,10,11,12,13,14,15].

Poly(butylene terephthalate) (PBT) and its copolymers with poly(ethylene oxide) (e.g., poly(butylene terephthalate-co-poly(alkylene glycol) terephthalate) (PBTAT) derivatives) are biodegradable polymers that are easy to process into films, fibers and foams [16,17,18,19]. Scaffolds based on PBT and/or PBTAT have been demonstrated to be suitable substrates for the attachment and proliferation of chondrocytes, mammalian skeletal muscle cells [19], bone marrow stromal cells [18] and human mesenchymal stem cells [17] in vitro. Preclinical studies in various animal models showed that the degradation rate of scaffolds based on PBT and/or PBTAT were dictated by the precise composition of the polymer backbone, which suggests that it may be possible to tailor-make such materials for specific conditions or patients; and in mammals, PBTAT-based materials encouraged bone growth, which motivates the development of PBT-/PBTAT-based scaffolds for bone regeneration [20,21,22,23].

Silk protein-based materials are also candidates for the generation of tissue scaffolds [24,25,26,27,28,29,30,31]. The natural silk fibroin of the domesticated Bombyx mori silkworm is the most commonly investigated for such applications [24,25,26,27,28,29,30,31,32]; however, recombinantly-produced silk-inspired proteins represent interesting alternatives because it is possible to produce large quantities of such silks with designed primary sequences [33,34,35,36,37]. Silk-based composites are also widely investigated for application as tissue scaffolds [37,38,39,40], and preclinical trials in animal models are promising [35,36,41].

Scheibel and coworkers have developed engineered spider silks based on the two most abundant proteins found in the dragline silks of the European garden spider (*Araneus diadematus*, *A. diadematus* fibroin 3 and 4, ADF3 and ADF4, respectively); the engineered silk protein analogues (eADF3 and eADF4, respectively) can be produced by an industrially-viable fermentation process in *Escherichia coli* bacteria [42,43,44,45]. The repetitive backbone sequence of eADF4 analogues displays numerous glutamic acid residues [42] enabling their chemical modification [46] or binding cations, such as drugs [47].

This manuscript describes the preparation and characterization of composites of PBT or PBTAT with an eADF4 analogue, namely eADF4(C16), and their biocompatibility as assayed with fibroblasts (M-MSV-BALB/3T3) and human mesenchymal stem cells. Moreover, mineralization of these composites with calcium phosphate enhanced the levels of alkaline phosphatase activity of human mesenchymal stem cells cultured on the substrates, and therefore, they are potentially useful for integration in biodegradable devices applied in bone tissues [48]. Such materials have prospects for application in tissue engineering and regenerative medicine, for use in various bone tissue-specific niches.

## 2. Materials and Methods

### 2.1. Materials

Unless otherwise stated, all chemicals were of ACS grade, purchased from Sigma-Aldrich Chemie GmbH (Schnelldorf, Germany) and used as supplied. Reagents for cell culture were purchased from Invitrogen (Carlsbad, CA, USA), unless otherwise noted. Human mesenchymal stem cells (HMSCs) were purchased from Lonza Cologne GmbH (Cologne, Germany). High glucose Dulbecco’s Modified Eagle Medium (DMEM) and fetal bovine serum (FBS) were purchased from Biochrom AG (Berlin, Germany). The recombinantly produced silk protein was based on the consensus motif of the repetitive core domain of one of the major ampullate silk fibroins of the garden cross spider (*A. diadematus* fibroin 4). The recombinant protein is composed of sixteen repeats of the polypeptide module C (amino acid sequence: GSSAAAAAAAASGPGGYGPENQGPSGPGGYGPGGP) and is referred to hereafter as eADF4(C16). Production and purification of eADF4(C16) were carried out as described previously [42].

### 2.2. Film Preparation, Thermogravimetric Analysis, X-ray Diffraction, Fourier Transform Infrared Spectroscopy, in Vitro Degradation Studies and in Vitro Fibroblast Adhesion Studies

Adapted from the previously described methodology [47], for the full experimental details refer to the Supplementary Materials.

### 2.3. Mineralization of Films with Calcium Carbonate

Three beakers (10 mL) containing crushed ammonium carbonate were also covered with parafilm punched with three needle holes and placed at the bottom of a large desiccator, above which films cast in 24-well tissue culture plates were incubated in an aqueous solution (1 mL) of calcium chloride (25 mM) and covered with parafilm punched with three needle holes. The desiccator was sealed and the samples left for 72 h. The samples were subsequently washed with water until the pH was neutral and then with ethanol/water (70% ethanol, 30% water) and allowed to dry in a sterile fume hood overnight.

### 2.4. Mineralization of Films with Calcium Phosphate

Films cast in 24-well tissue culture plates were incubated in an aqueous solution (1 mL) of calcium chloride (200 mM) for 20 min, after which the solution was removed, and the samples were washed with water (3 × 1 mL). Thereafter, samples were incubated in an aqueous solution (1 mL) of sodium phosphate (120 mM) for 20 min, after which the solution was removed, and the samples were washed with water (3 × 1 mL). The cycle of incubation with calcium chloride and sodium phosphate was repeated a further six times (i.e., a total of 7 cycles), after which the samples were incubated in ethanol/water (70% ethanol, 30% water) for 30 min and allowed to dry in a sterile fume hood overnight.

### 2.5. Scanning Electron Microscopy and Energy Dispersive Spectroscopy

Samples were mounted on metal stubs, coated with Pt/Pd or carbon using a Cressington 208 benchtop sputter coater (Redding, CA, USA) before being observed with a Hitachi S5500 SEM equipped with an EDS probe (Mannheim, Germany).

### 2.6. Stem Cell Culture and Qualitative and Quantitative Studies of Alkaline Phosphatase Activity

Commercially available Nunclon^®^ Δ surface (Thermo Fisher Scientific, Nidderau, Germany) tissue culture plates were used for control experiments. Silk films were sterilized by incubation in 70% ethanol solution followed by exposure to UV for 60 min. After sterilization, the samples were incubated for 30 min under 3 mm of HMSC growth medium. The HMSC growth medium was composed of: high glucose Dulbecco’s Modified Eagle Medium (DMEM, 440 mL); fetal bovine serum (50 mL); antibiotic-antimycotic (5 mL); non-essential amino acids (5 mL); and 2 ng/mL basic fibroblast growth factor. Medium was aspirated and replaced prior to HMSC seeding. Cell viability before starting the experiment was determined by the Trypan Blue exclusion method, and the measured viability exceeded 95% in all cases. HMSCs were seeded at 10,000 cells/cm^2^ under 3 mm of medium and incubated at 37 °C, 95% humidity and a CO_2_ content of 5%. After 3 days, the medium was aspirated; the films were washed gently with phosphate buffered saline (PBS) and replaced with osteogenic medium. Osteogenic medium was composed of: high glucose Dulbecco’s Modified Eagle Medium (DMEM, 425 mL); fetal bovine serum (50 mL); antibiotic-antimycotic (5 mL); non-essential amino acids (5 mL); dexamethasone (100 nM); β-glycerol phosphate (10 mM); and ascorbic acid (50 µM). Thereafter, the osteogenic medium was aspirated and replaced every 2 days until the samples were analyzed. Alkaline phosphatase (ALP) activity was visualized with a Leukocyte Alkaline Phosphatase Kit (Sigma-Aldrich Chemie GmbH, (Schnelldorf, Germany)) using the manufacturer’s protocol. Images of stained cells were obtained using a camera AxioCam MRm attached to a Zeiss Axio Observer Z1 equipped with an ApoTome unit. Images are representative of 3 samples. DNA was quantified using PicoGreen^®^ assay (Life Technologies GmbH, Darmstadt, Germany) using a Synergy HT Multi-Mode Microplate Reader (Bio-tek Instruments GmbH, Bad Friedrichshall, Germany). ALP activity of the cell population was quantified by first scraping and breaking up the films in a buffer of 0.2% Triton X-100 (Sigma-Aldrich Chemie GmbH (Schnelldorf, Germany)) and then measuring ALP activity using an ALP LiquiColor^®^ kit (Stanbio, Boerne, TX, USA) in accordance with the manufacturer’s protocol. The sample and reagents were incubated in a 96-well plate for 1 h at 37 °C and then read using a Synergy HT Multi-Mode Microplate Reader (Bio-tek Instruments GmbH, Bad Friedrichshall, Germany). Data were normalized to DNA quantity. Statistical analysis via ANOVA (null hypothesis that all groups have the same true mean, *p*-value < 0.0001) was carried out within R [49], and one-way ANOVA statistics were calculated and interpreted with Tukey’s *t*-test, for which any interval that does not cross zero (the dashed line) is significant with an alpha = 0.05 [9].

## 3. Results and Discussion

### 3.1. Film Preparation and Characterization

The compositions of the films described herein are found in Table 1. All films had a thicknesses of ca. 100 µm and therefore would not be expected to be encapsulated inside a very thick foreign body capsule in vivo [47]. Thermogravimetric analysis revealed that “as-cast” films contained residual volatiles (1,1,1,3,3,3-hexafluoroisopropanol (HFIP) and water), the levels of which were diminished by immersion of the films in methanol (Figures S1–S9, Supplementary Materials).

Analysis of the films by X-ray diffraction (Figures S1–S9 and Table S1) was informative, confirming that the eADF4(C16) silk component of the “as-cast” films was water soluble due to its α-helix-rich nature (XRD peaks at 2θ = 14.4° and 19.4°) induced by the HFIP used in the casting process [47] and that methanol treatment rendered the silk component of films insoluble in water due to the induction of β-sheet formation (XRD peaks at 2θ = 16.7°, 19.9°, 24.0° and 31.8°, in agreement with literature data), suggesting that this process removes residual HFIP [47]. The peak positions for PBT [50,51] or PBTAT [50,51] are in line with those reported in the literature for each polymer, respectively. Interestingly, the XRD spectra of the films composed solely of PBT or PBTAT revealed that they became more crystalline after treatment with methanol, which supports our assertion that methanol treatment removes residual HFIP that solvates the polymers, thereby deterring their crystallization. XRD spectra of films composed of mixtures of eADF4(C16) and the PBT or PBTAT displayed peaks due to the combinations of the two components; however, the signals of eADF4(C16) were normally only evident as shoulders on the peaks due to the more crystalline PBT or PBTAT.

FTIR spectroscopy confirmed that HFIP (Figure S10) was present in the “as-cast” films (strong absorption at 1105 cm^−1^) and that it could effectively be removed by methanol treatment, as the absorption was markedly diminished or absent (Figures S1–S9). Furthermore, FTIR spectroscopy confirmed the silk component of the as-cast films to be α-helix rich (amide I and II peaks were observed at 1656 and 1547 cm^−1^, respectively), whereas the methanol-treated films were β-sheet rich (amide I and II absorptions were shifted to 1625 and 1521 cm^−1^ respectively, and a peak at 965 cm^−1^ was assigned to polyalanine-based β-sheets).

Visual observation of the “as-cast” and “methanol treated” films by photography and bright field microscopy (Figures S1–S9) revealed a degree of phase separation between the eADF4(C16) and PBT or PBTAT (analogous to that observed for composites of eADF4(C16) and polycaprolactone or Pellethane 2363-80A) [47]. Differences in the optical properties of the components of the films (the silk being relatively clear and the PBT/PBTAT being relatively opaque) enabled the assignment of the component constituting the continuous phase as reported in Table 1.

### 3.2. In Vitro Degradation Studies

A biomaterial’s performance in vivo is influenced by its stability and degradation profile. For tissue engineering applications, materials that degrade are attractive as they can be replaced by native extracellular matrix, and it is useful to be able to tune the degradation behavior of biomaterials [24,32,52]. Trypsin and elastase were chosen as biologically-relevant model proteolytic enzymes that play roles in digestion and wound healing, respectively. The in vitro degradation of the films in solutions of elastase and trypsin in phosphate buffered saline (PBS) was studied over the period of 250 h (Figures S1–S9). Spontaneous hydrolysis of eADF4(C16), PBT and PBTAT has been reported to be negligible (<2%), as they are insoluble in water, and hydrolysis of the amides and esters in their respective backbones is a very slow process [22,23,24,47]. In the presence of elastase and trypsin, the films composed solely of eADF4(C16) were observed to degrade slowly and had sufficient structural integrity to be manipulated for over 250 h (Figure S1). Mass loss profiles recorded using the same procedure for PBT-25 (Figure S2) and PBTAT-25 (Figure S6) films showed that they degraded more swiftly, in part because their phase separated nature formed the basis for small parts of the film separating from the bulk; their degradation profiles are included for completeness and not representative solely of the enzymatic degradation of the silk protein. The structural integrity of all of the other films was maintained for the duration of the experiments, and the data are therefore representative of the enzymatic degradation of the silk protein; mass loss was faster from films with higher eADF4(C16) content. Clearly, it would be expected that the degradation of the films in vivo would be markedly slower than that of our in vitro assay, in line with the literature precedent for *Nephila clavipes* spider silk [53], *B. mori* silkworm silk [41] or the polyesters [22,23], respectively.

### 3.3. In Vitro Fibroblast Adhesion Studies

BALB/3T3 mouse fibroblast adhesion to the films was assayed using Alamar Blue, with two commercially available surfaces as references for our studies, untreated polystyrene tissue culture plates (Nunclon^®^) and plasma-treated polystyrene tissue culture plates (Nunclon^®^ Δ Surface), and cell adhesion is reported relative to the Nunclon^®^ Δ surface [46,47]. Since the cells were in a quasi-steady-state situation, increasing values of fluorescence are proportional to the number of cells, observing fibroblast adhesion on all of the films (Table 1 and Supplementary Materials). Fibroblast adhesion to films incorporating PBT or PBTAT was in all cases better than to films composed of eADF4(C16) alone (which already have been described to be a poor surface for fibroblast adhesion), and generally comparable to levels of adhesion observed for the untreated Nunclon^®^ tissue culture plates; interestingly, levels of cell adhesion to PBTAT-50 films were similar to that on plasma-treated Nunclon^®^ Δ surface tissue culture plates. Cells were clearly observable on the optically clear films of eADF4(C16) and tissue culture plates (Figures S1, S11 and [47], respectively), whereas cells on the composite films were more easily visualized after calcein A/M staining (Figures S2–S9).

### 3.4. Film Biomineralization with Calcium Carbonate or Calcium Phosphate

**Figure 1:**
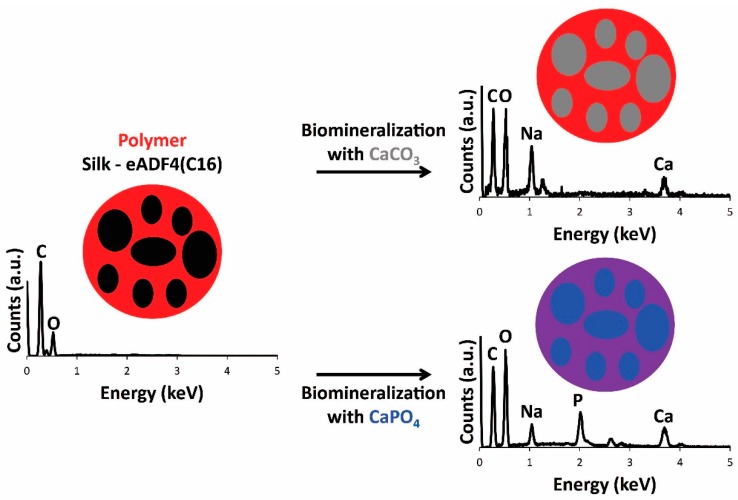
Schematic of the biomineralization of films with representative EDS analysis of films.

**Figure 2:**
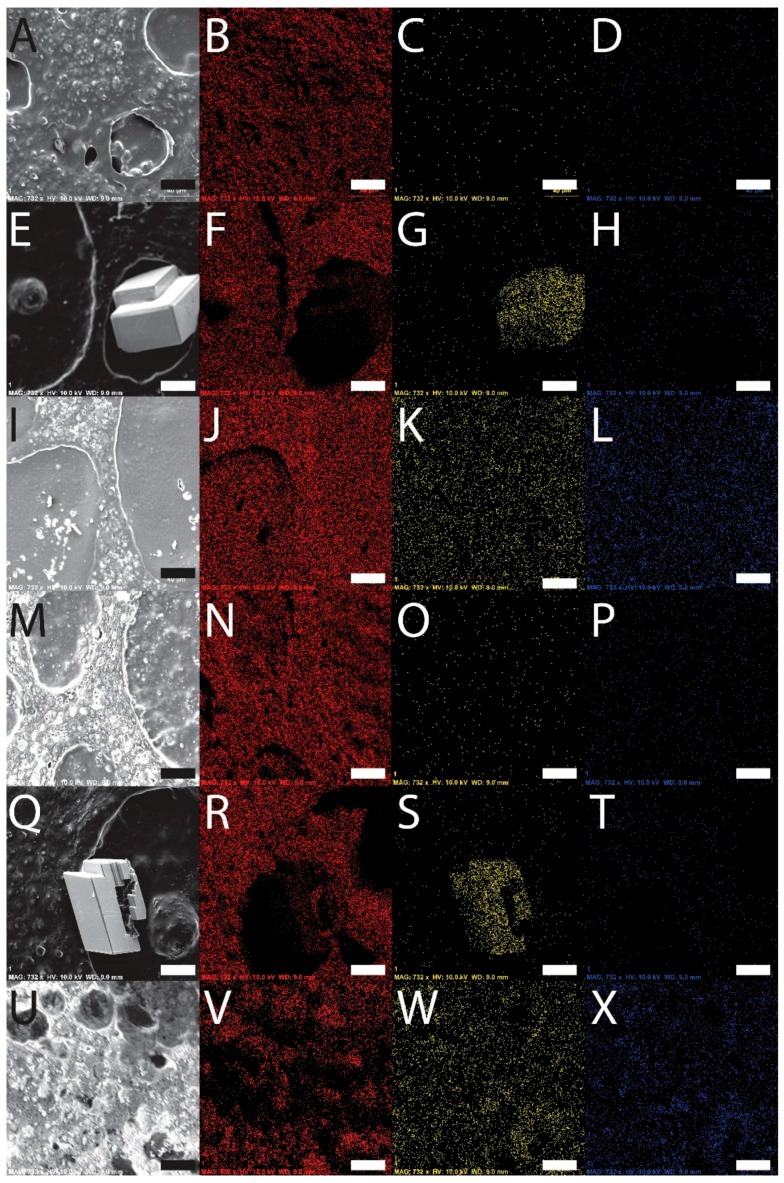
SEM-EDS analysis of films. (**A**–**D**) PBT-50; (**E**–**H**) PBT-50-CaCO_3_; (**I**–**L**) PBT-50-CaPO_4_; (**M**–**P**) PBTAT-50; (**Q**–**T**) PBTAT-50-CaCO_3_; (**U**–**X**) PBTAT-50-CaPO_4_; (**A**,**E**,**I**,**M**,**Q**,**U**) secondary electron SEM image; (**B**,**F**,**J**,**N**,**R**,**V**) carbon, red; (**C**,**G**,**K**,**O**,**S**,**W**) calcium, yellow; (**D**,**H**,**L**,**P**,**T**,**X**) phosphorous, blue. The scale bar represents 40 µm.

With a view toward the application of the materials as scaffolds for bone tissue engineering, the films were biomineralized [54,55] with calcium carbonate or calcium phosphate. Mineralization of the films with calcium carbonate was achieved by incubation of the films in solutions of calcium chloride in a container with ammonium carbonate, and mineralization of the films with calcium phosphate was achieved by iterative sequences of incubation of the films in solutions of calcium chloride followed by sodium phosphate. The engineered silk eADF4(C16) displays multiple carboxylic acid moieties capable of binding calcium ions, facilitating their mineralization. Energy dispersive spectroscopy (EDS) analysis of the films confirmed that the surface chemistry of the films before and after mineralization was different. Peaks in the EDS spectra of the eADF4(C16) and composite films prior to mineralization have lines at 0.277, 0.525 and 1.041 keV that are the characteristic Kα emissions of carbon, oxygen and sodium, respectively, and the weak emission at 0.392 keV is the Kα emission of nitrogen (Figure 1). After the mineralization, new peaks appeared in the spectra at 2.013, 2.621 and 3.690 keV, which are the characteristic Kα emission lines of phosphorous, chlorine (from the calcium chloride used as a source of Ca^2+^) and calcium, respectively (Figure 1). Imaging with SEM-EDS revealed that calcium carbonate was preferentially deposited in the eADF4(C16) phase of the films, as opposed to the PBT or PBTAT phases, whereas the calcium phosphate was deposited more homogeneously across the surface of the films (as depicted in schematic format in Figure 1); this is likely to be caused by differences in the concentration of calcium chloride solution in which the films were incubated, 25 mM for calcium carbonate mineralization as opposed to 200 mM for calcium phosphate deposition (examples for PBT-50 and PBTAT-50 are displayed in Figure 2).

### 3.5. In Vitro Stem Cell Culture

**Figure 3:**
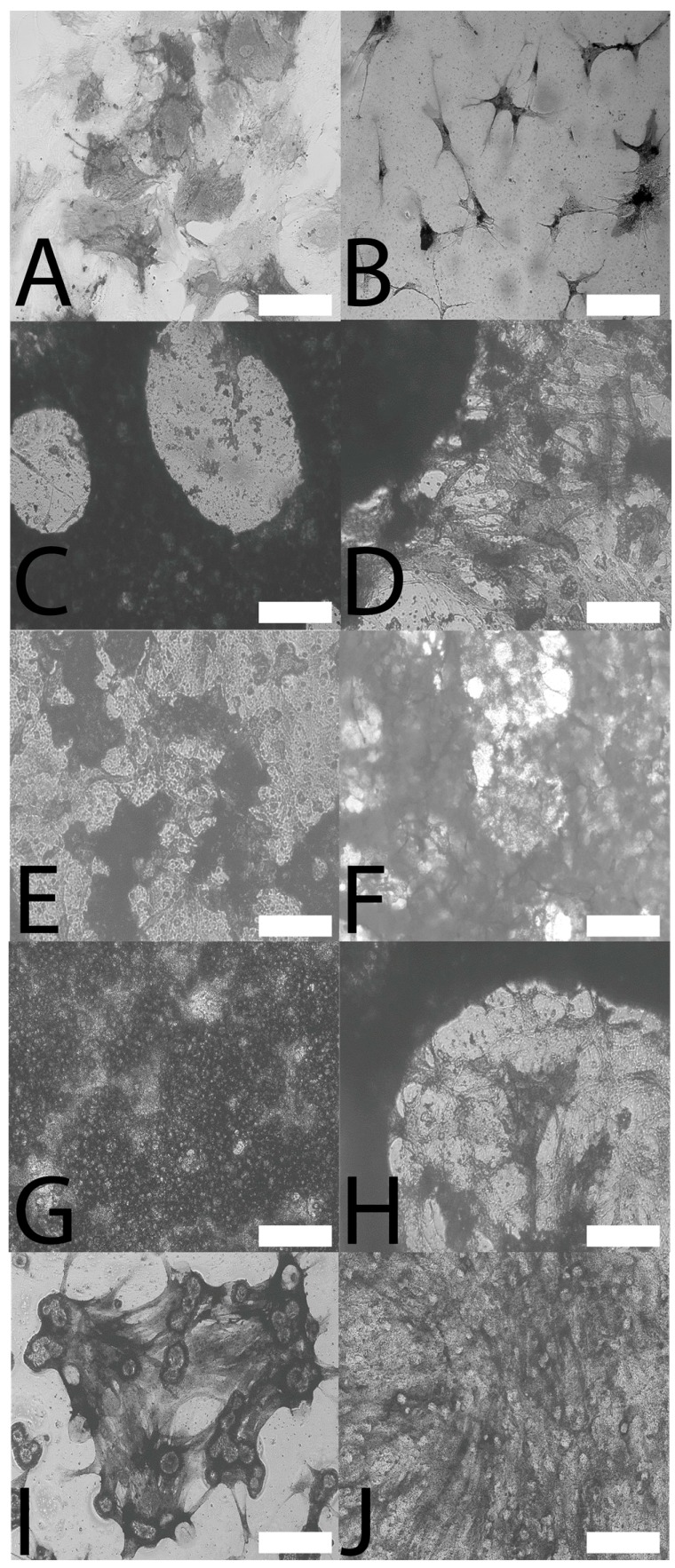
(**A**–**J**) Qualitative analysis of the ALP activity of stem cells on films mineralized with calcium phosphate using bright field microscopy after ALP live staining; (**A**) Nunclon^®^ Δ; (**B**) eADF4(C16)-CaPO_4_; (**C**) PBT-25-CaPO_4_; (**D**) PBT-50-CaPO_4_; (**E**) PBT-75-CaPO_4_; (**F**) PBT-100-CaPO_4_; (**G**) PBTAT-25-CaPO_4_; (**H**) PBTAT-50-CaPO_4_; (**I**) PBTAT-75-CaPO_4_; (**J**) PBTAT-100-CaPO_4_. Scale bars represent 200 µm.

**Figure 4:**
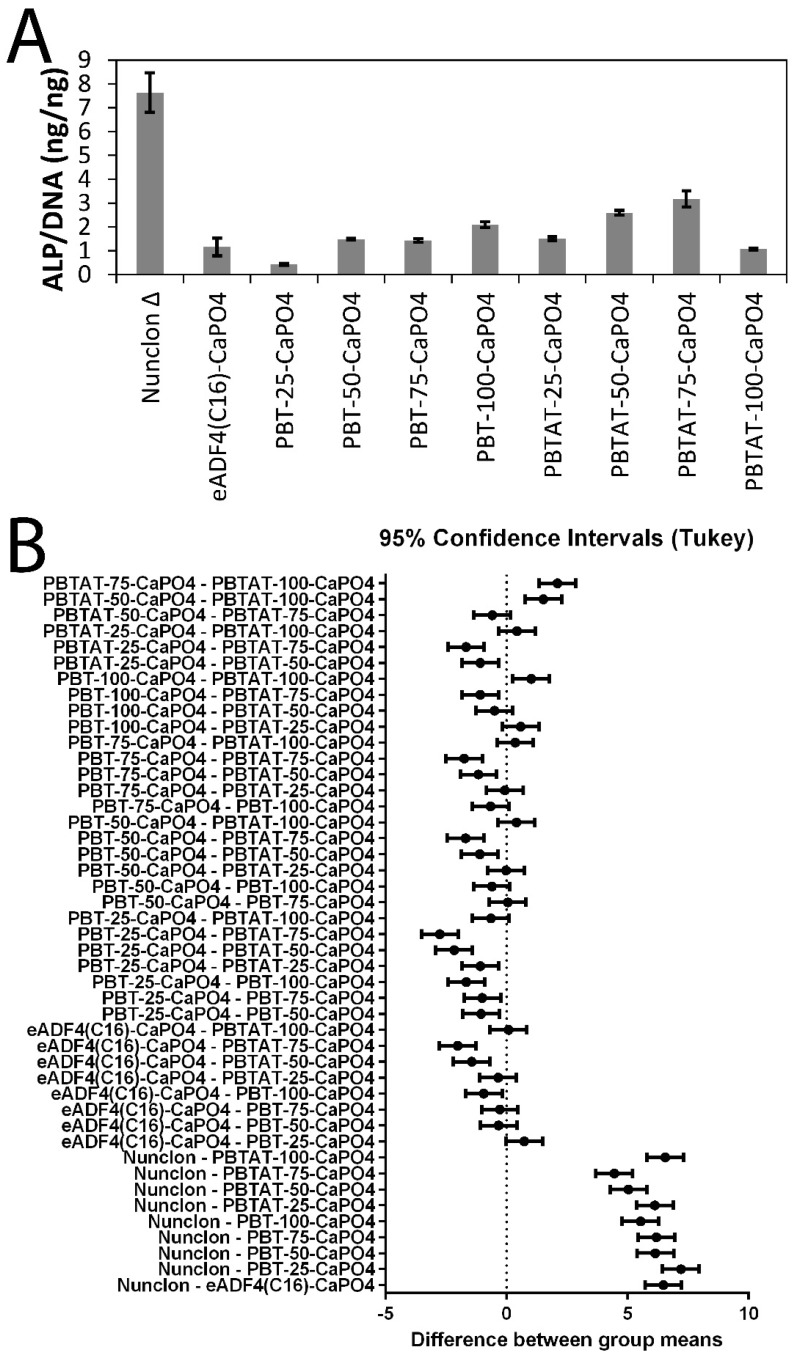
(**A**) Quantitative analysis of the ALP activity of stem cells on films mineralized with calcium phosphate; (**B**) statistical analysis via ANOVA (null hypothesis that all groups have the same true mean, *p*-value < 0.0001), and one-way ANOVA statistics were calculated and interpreted with Tukey’s *t*-test, for which any interval that does not cross zero (the dashed line) is significant with an alpha = 0.05.

Human mesenchymal stem cells were cultured in vitro for two weeks on calcium phosphate mineralized films. Alkaline phosphatase (ALP) activity is a hallmark of bone tissue formation, and therefore, both qualitative and quantitative analyses of ALP activity were studied. Qualitative analysis of ALP activity using ALP live staining (Figure 3A–J) showed that the cells were alive and functional on the films as seen by the patches of dark coloration that are characteristic of the precipitated stain. Quantitative analysis of ALP activity for the cells cultured on the mineralized films (Figure 4) showed that ALP activity (Figure 4A) was correlated with levels of fibroblast adhesion (Table 1). The one-way analysis of variance (ANOVA) was used to determine whether there were any significant differences in the quantitative analyses of ALP activity (Figure 4B), and the one-way ANOVA rejects the null hypothesis that all groups have the same true mean (*p*-value < 0.0001). Consequently, Tukey’s *t*-test was used to compare differences between groups, where any interval that does not cross zero (the dashed line in Figure 4B) is significant with an alpha = 0.05. Interestingly, levels of ALP activity for the cells cultured on Nunclon^®^ Δ were significantly different from all other films. Levels of ALP activity for the cells cultured on mineralized eADF4(C16) were not significantly different from the mineralized PBT composites or, indeed, the pure PBT or PBTAT; however, statistically-significant differences were observed for mineralized PBTAT-50 and PBTAT-75, wherein ALP activity for cells cultured on these materials was higher than for either of the constituents (eADF4(C16) or PBTAT) alone (and logically, the PBT composites). Together, this suggests that composites of eADF4(C16) and PBTAT have some potential for bone tissue engineering.

## 4. Conclusions

Films composed of natural and recombinantly-produced silk proteins have been widely investigated for biomedical applications, such as biocompatible coatings for biomedical implants, owing to the facility with which silk proteins can be processed into films with tunable surface properties (morphology, hydrophilicity, etc.), their biodegradability and low levels of immunogenicity in vitro/in vivo. This manuscript reports a simple method of producing films composed of a recombinantly-produced spider silk inspired protein eADF4(C16) and biodegradable polymers (PBT and PBTAT), their mineralization with either calcium carbonate or calcium phosphate and a preliminary in vitro cell culture experiment to assess their efficacy for bone tissue engineering. Interestingly, levels of ALP activity for HMSCs residing on calcium phosphate-mineralized PBTAT-50 and PBTAT-75 films were elevated when compared to the other formulations investigated or indeed the constituents alone, and it is concluded that such composites have potential for the development of functional biomineralized biomaterials [56,57,58,59,60,61,62,63].

## Figures and Tables

**Table 1:** Film compositions and properties. PBTAT, poly(butylene terephthalate-*co*-poly(alkylene glycol) terephthalate).

Film	Mass Ratio Protein:Polymer	Continuous Phase	Fibroblast Adhesion Relative to Nunclon^®^ Δ Surface (%)	Figure
eADF4(C16)	100:0	eADF4(C16)	72.0 ± 8.0	S1 and [7]
PBT-25	75:25	eADF4(C16)	55.5 ± 5.9	S2
PBT-50	50:50	PBT	58.9 ± 8.0	S3
PBT-75	25:75	PBT	69.8 ± 10.0	S4
PBT-100	0:100	PBT	75.8 ± 3.5	S5
PBTAT-25	75:25	eADF4(C16)	76.9 ± 6.6	S6
PBTAT-50	50:50	PBTAT	104.5 ± 4.4	S7
PBTAT-75	25:75	PBTAT	76.4 ± 2.4	S8
PBTAT-100	0:100	PBTAT	69.3 ± 2.4	S9
Untreated Nunclon^®^	Not applicable	Not applicable	74.0 ± 6.2	S11
Nunclon^®^ Δ Surface	Not applicable	Not applicable	100.0 ± 7.5	[47]

## Supplementary Materials

The following are available online at www.mdpi.com/1996-1944/9/7/560/s1. Figure S1: eADF-4(C16) films. Figure S2: PBT-25 films. Figure S3: PBT-50 films. Figure S4: PBT-75 films. Figure S5: PBT-100 films. Figure S6: PBTAT-25 films. Figure S7: PBTAT-50 films. Figure S8: PBTAT-75 films. Figure S9: PBTAT-100 films. Table S1: Positions of XRD peaks of films determined using Jade 9 XRD Pattern Processing software. Figure S10: FTIR spectrum of pure HFIP. Figure S11: Bright field microscope image of fibroblasts cultured on Nunclon^®^ Tissue Culture Plate (scale bar represents 100 µm).
