# Supplementary material for: Biomineralization of Engineered Spider Silk Protein-Based Composite Materials for Bone Tissue Engineering
Source: Materials (Basel). 2016 Jul 11;9(7):560. doi: 10.3390/ma9070560 (PMC5456849; doi:10.3390/ma9070560)
Supplement: Supplementary file 1 [file materials-09-00560-s001.pdf]

Supplementary Materials: Biomineralization of Engineered Spider Silk Protein-Based Composite Materials for Bone Tissue Engineering

John G. Hardy, Jose Guillermo Torres-Rendon, Aldo Leal-Egaña, Andreas Walther, Helmut Schlaad, Helmut Cölfen and Thomas R. Scheibel

Supplementary Materials and Methods

Film Preparation

Optically clear solutions of PBT, PBTAT and eADF4(C16) dissolved in various ratios in 1,1,1,3,3,3-hexafluoroisopropanol (HFIP) were cast in 24 well Nunclon® Δ surface tissue culture plates (Thermo Fisher Scientific, Nidderau, Germany) or on flexible Teflon substrates. The solvent was allowed to evaporate over a period of 24 h in a fume hood, and the samples are hereafter referred to as "as cast". Films that were subsequently immersed in anhydrous methanol for 1 h prior to drying for a further 48 h under high vacuum are hereafter referred to as "methanol treated". The thickness of the films was determined with high precision digital calipers (Bochem, Weilberg, Germany). The proteins and polymers were phase separated in the films. Optical microscopy (Leica DFC295 camera mounted on an inverse microscope, Leica DMIL LED microscope, Wetzlar, Germany) was used to determine the component constituting the continuous phase of the films through differences in optical properties.

Thermogravimetric Analysis (TGA)

Analyses were carried out with a Mettler Toledo TGA/SDTA 851E thermobalance (Mettler Toledo GmbH, Giessen, Germany). Films were precisely weighed into ceramic crucibles (VWR, Darmstadt, Germany), and analyses were carried out under a nitrogen atmosphere (flow rate 100 mL per minute), over a temperature range between 25 and 800 °C, at a heating rate of 10 °C/min. The TGA mass loss profiles are representative of at least 2 samples. Adapted from previously described methodology [47].

X-ray Diffraction (XRD)

A stack of 3 films was attached to a metal sample holder using adhesive tape. XRD spectra were recorded on a Bruker D8 Advance X-ray diffractometer (Frankfurt, Germany, CuKα1-beam, λ = 154,051 pm, recording angle 6°–40° (2θ), angle increment 0.1° (2θ), recording time of 1 min per angle position). The XRD patterns (plotted as intensity vs. 2θ) were analyzed using Jade 9 XRD Pattern Processing software (Materials Data Inc., Livermore, CA, USA), and the d-spacings reported have an accuracy typically in the range of ±2%. Adapted from previously described methodology [47].

Fourier Transform Infrared (FTIR) Spectroscopy

FTIR spectra of films were recorded by attenuated total reflection (ATR) on a Bruker Tensor 27 spectrometer equipped with a Ge crystal (Bruker, Frankfurt, Germany). Scan range: 900–1800 cm^{-1}, resolution of 1 cm^{-1}, 8 scans. Adapted from previously described methodology [47].

In Vitro Degradation Studies

Films (of ca. 20 mg) were stored for 1 week under high vacuum at 21 °C, after which time their weight was determined on a high precision balance. The films were incubated at 37 °C in 1.5 mL phosphate buffered saline (PBS) at a pH of 7.4 in the presence of elastase (0.8 μg) and trypsin (12.5 μg); control samples were incubated at 37 °C in 1.5 mL PBS without enzyme. At specific time points the buffer was removed, the films were carefully washed twice with 1.5 mL of deionized water, and then dried under high vacuum at 21 °C for 72 h, after which their weight was

determined on a high precision balance. The films were subsequently placed in a fresh solution of buffer (with or without enzymes), and their weight was followed over a period of 250 h in total. The mass loss profiles are the average of at least 3 samples. Adapted from previously described methodology [47].

Fibroblast Adhesion Studies

The fibroblast cell line (M-MSV-BALB/3T3, mouse embryo fibroblasts) was sourced from the European Collection of Cells (Salisbury, UK). The cells were cultivated in DMEM (Biochrom AG, Berlin, Germany) supplemented with 10% *v*/*v* fetal bovine serum (BioChrom AG, Berlin, Germany), 2 mM Glutamax (Gibco, Darmstadt, Germany) and 50 µg/mL Gentamycin (Sigma, Schnelldorf, Germany). Cells were cultured on films cast on 24 well plates (Nunclon® Δ surface). The viability observed for the cells before starting the experiment was determined by the Trypan Blue (Sigma, Schnelldorf, Germany) exclusion method, and the measured viability exceeded 95% in all cases. Cell adhesion was determined with the AlamarBlue® cell viability assay (Cell Titer-Blue, Promega, Madison, WI, USA) according to the protocol of the supplier. Briefly, 100,000 cells/cm^2 were seeded in wells, and the cells were incubated in 1 mL of cell culture media per well at 37 °C, 95% humidity, and a CO_2 content of 5%. After 4 h the cells were washed gently with PBS to remove non-adherent and/or dead cells, followed by the addition of fresh media (1 mL) containing 10% *v*/*v* of the AlamarBlue® reagent. After 2.5 h of culture, 100 µL of the media containing the AlamarBlue® reagent was removed and placed in a 96 well plate, and the fluorescence was measured with a fluorimeter (Mithras LB 940, Berthold Technologies, Bad Wildbad, Germany). Two controls were considered during the measurement of the fluorescence: the first was wells containing media alone (i.e., no cells or AlamarBlue® reagent), which was not fluorescent (data not shown); and the second was wells containing the AlamarBlue® reagent but no cells (used for baseline correction). Levels of cell adhesion to the various films studied herein are reported relative to Nunclon® Δ surface, which was assigned an arbitrary value of 100%. Commercially available untreated Nunclon® and plasma treated Nunclon® Δ surface tissue culture plates were used for control experiments. Experimental films were sterilized by incubation in 70% ethanol solution followed by exposure to UV for 60 min. Cells on transparent substrates were visualized by optical microscopy (Leica DFC295 camera mounted on an inverse microscope, Leica DMIL LED microscope, Wetzlar, Germany). Cells on non-transparent surfaces were stained with Calcein acetoxymethyl ester (Calcein A/M) (Invitrogen, Eugene, OR, USA) added to the medium at a final concentration of 2 mM, and incubated for 10 min at 37 °C, prior to visualization with a Leica DMI3000 B fluorescence microscope (Leica, Wetzlar, Germany). Images are representative of 3 samples. Adapted from previously described methodology [47].

Supplementary Figures and Tables

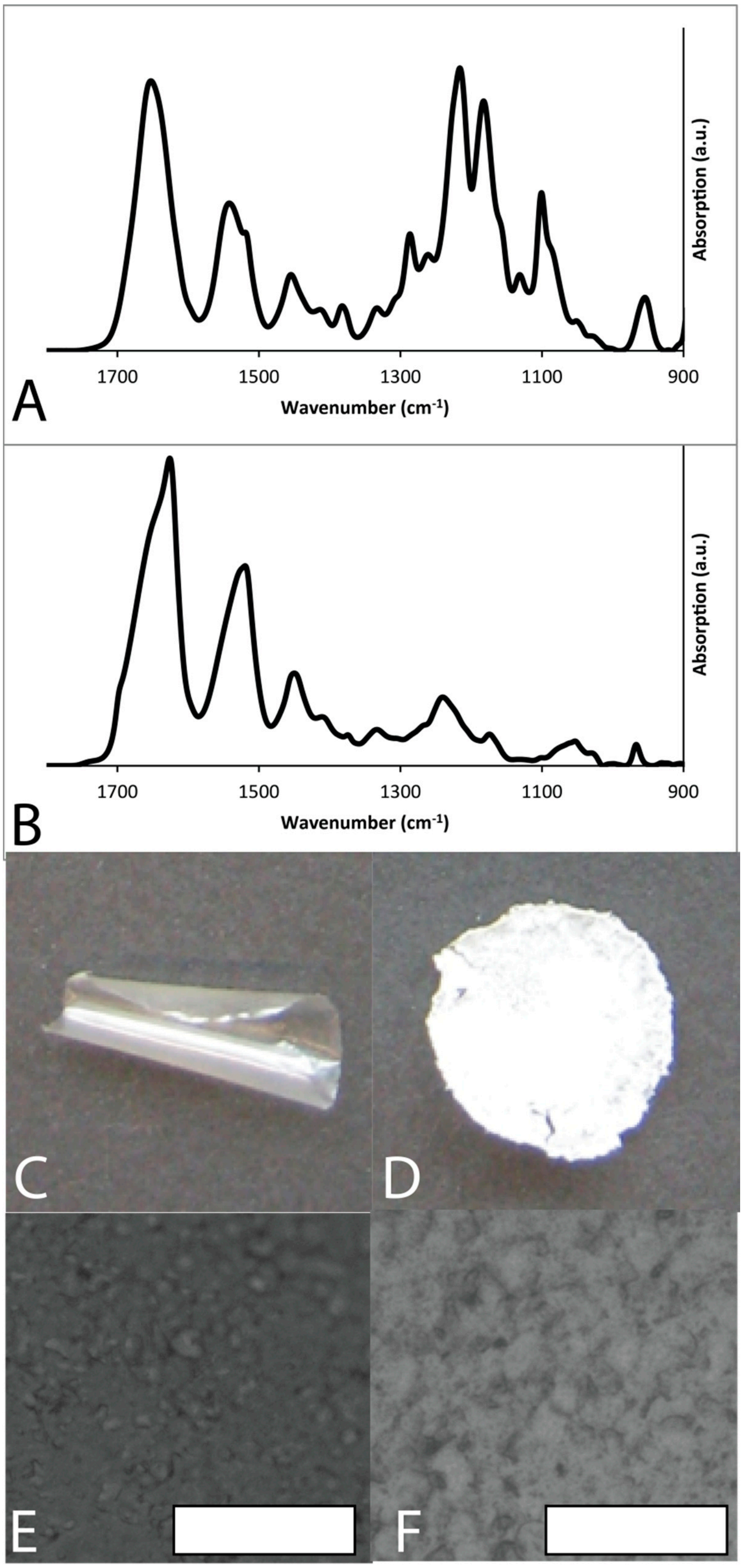

Figure S1. eADF-4(C_{16}) films. (**A**) FTIR spectra of as cast films; (**B**) FTIR spectra of films after methanol treatment; (**C**,**D**) Photographs of "as cast" and "methanol treated" films with diameter of ca. 17 mm, respectively; (**E**,**F**) Bright field microscope images of "as cast" and "methanol treated" films, respectively (scale bars represent 100 µm).

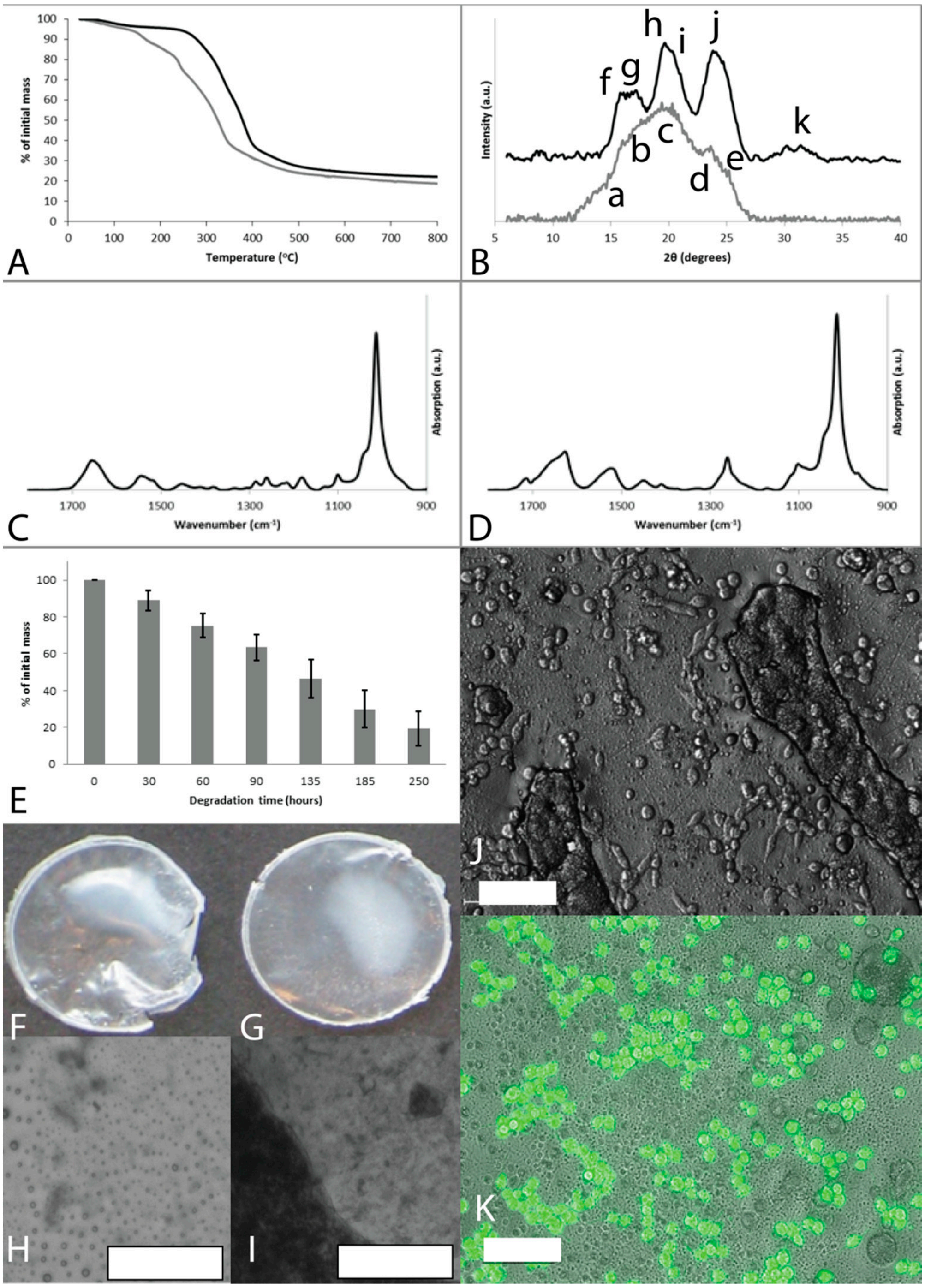

Figure S2. PBT-25 films. (**A**) TGA mass loss profiles, as cast films (grey line) and films after methanol treatment (black line); (**B**) XRD spectra, as cast films (grey line) and films after methanol treatment (black line); lowercase letters label peaks analyzed in Table S1; (**C**) FTIR spectra of as cast films; (**D**) FTIR spectra of films after methanol treatment (right); (**E**) Degradation of methanol treated films upon exposure to a combination of Elastase and Trypsin; (**F**) Photograph of as cast film with diameter of ca. 17 mm; (**G**) Photograph of methanol treated film with diameter of ca. 17 mm; (**H**) Bright field microscope image of as cast film (scale bar represents 100 μm); (**I**) Bright field microscope image of methanol treated film (scale bar represents 100 μm); (**J**) Bright field microscope image of mouse embryo fibroblasts cultured on methanol treated films for 6.5 h (scale bar represents 100 μm); (**K**) Fluorescence microscope image of Calcein A/M stained fibroblasts cultured on methanol treated films (scale bar represents 100 μm).

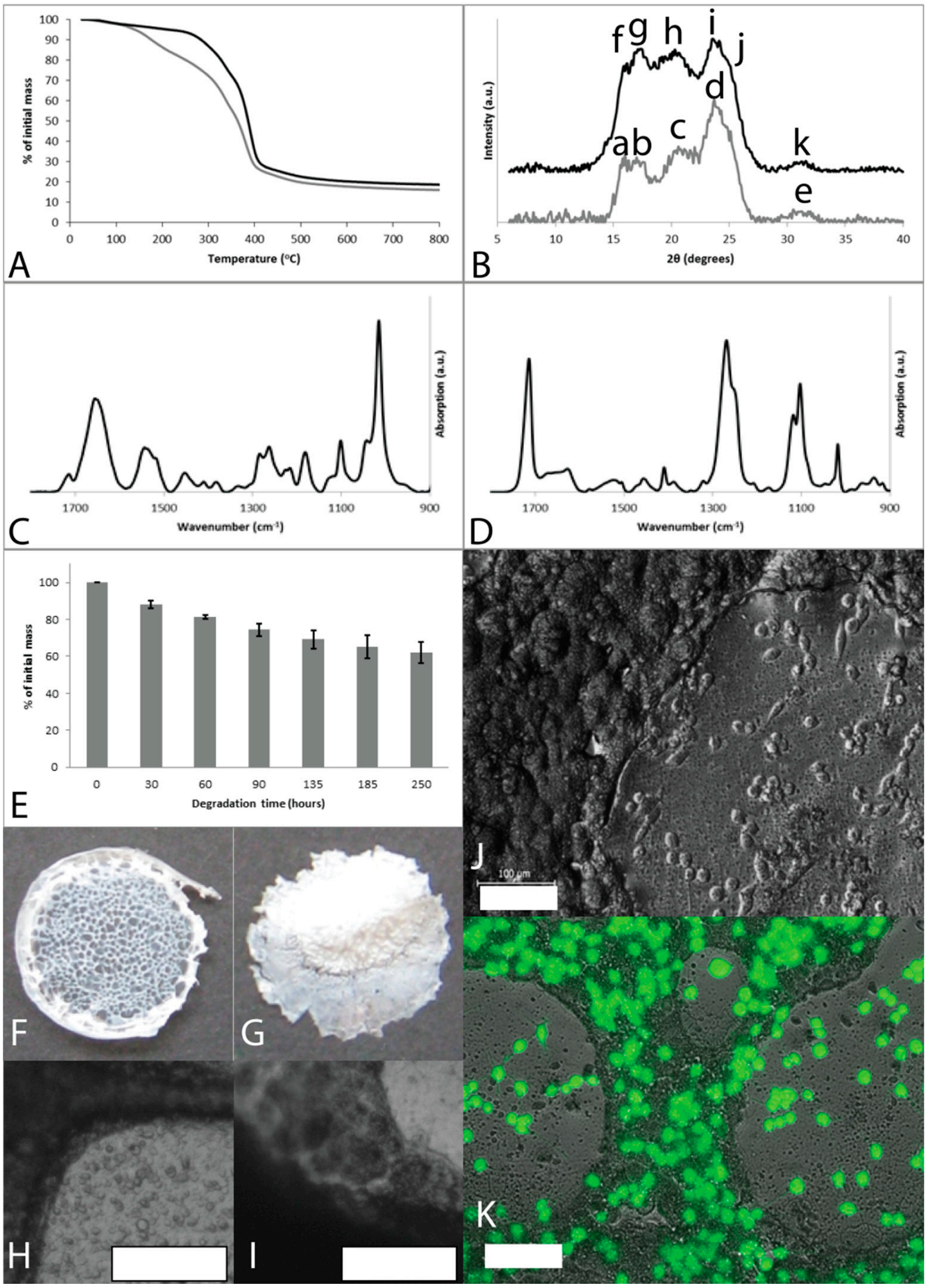

Figure S3. PBT-50 films. (**A**) TGA mass loss profiles, as cast films (grey line) and films after methanol treatment (black line); (**B**) XRD spectra, as cast films (grey line) and films after methanol treatment (black line); lowercase letters label peaks analyzed in Table S1; (**C**) FTIR spectra of as cast films; (**D**) FTIR spectra of films after methanol treatment (right); (**E**) Degradation of methanol treated films upon exposure to a combination of Elastase and Trypsin; (**F**) Photograph of as cast film with diameter of ca. 17 mm; (**G**) Photograph of methanol treated film with diameter of ca. 17 mm; (**H**) Bright field microscope image of as cast film (scale bar represents 100 μm); (**I**) Bright field microscope image of methanol treated film (scale bar represents 100 μm); (**J**) Bright field microscope image of mouse embryo fibroblasts cultured on methanol treated films for 6.5 h (scale bar represents 100 μm); (**K**) Fluorescence microscope image of Calcein A/M stained fibroblasts cultured on methanol treated films (scale bar represents 100 μm).

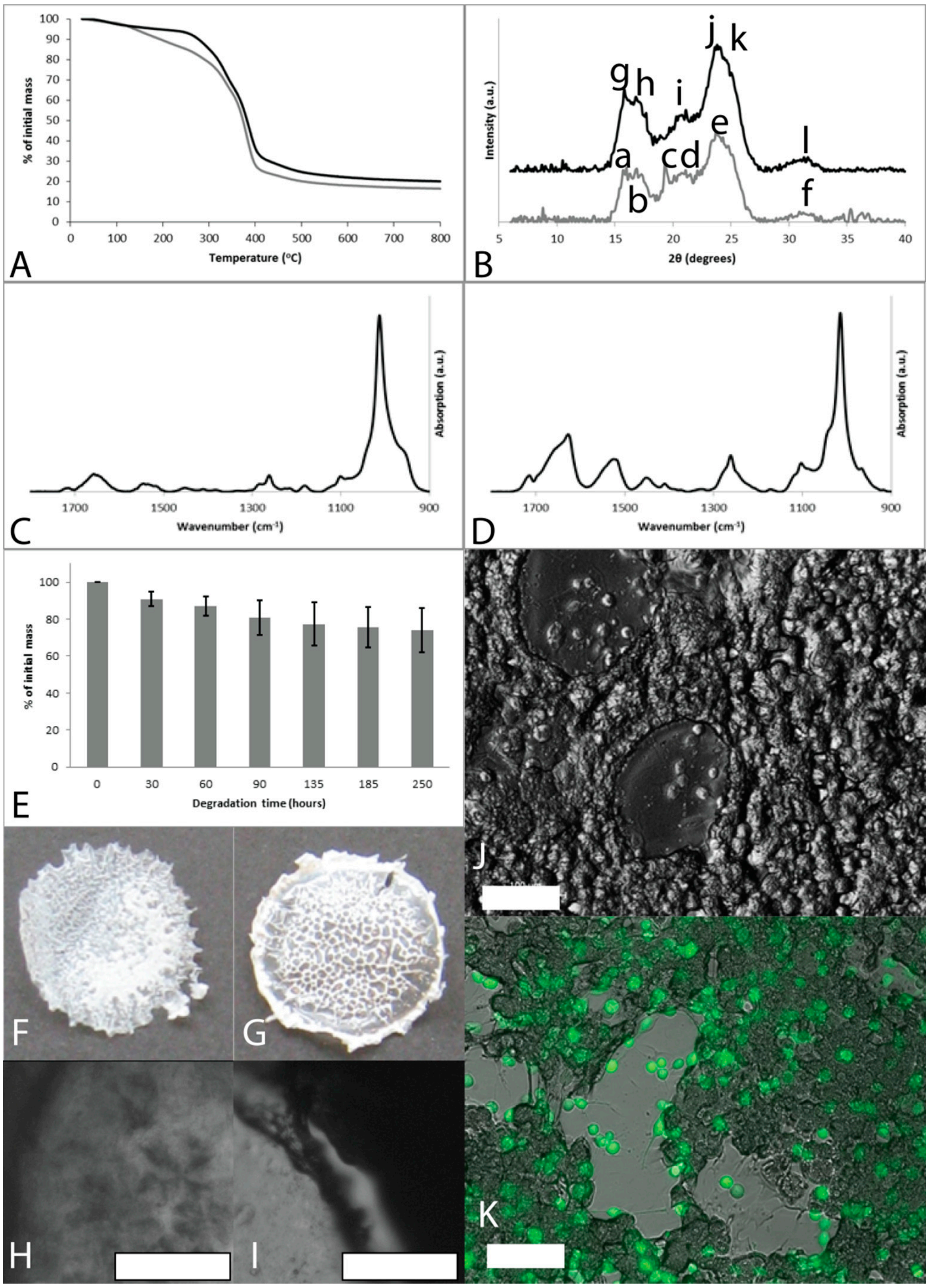

Figure S4. PBT-75 films. (**A**) TGA mass loss profiles, as cast films (grey line) and films after methanol treatment (black line); (**B**) XRD spectra, as cast films (grey line) and films after methanol treatment (black line); lowercase letters label peaks analyzed in Table S1; (**C**) FTIR spectra of as cast films; (**D**) FTIR spectra of films after methanol treatment (right); (**E**) Degradation of methanol treated films upon exposure to a combination of Elastase and Trypsin; (**F**) Photograph of as cast film with diameter of ca. 17 mm; (**G**) Photograph of methanol treated film with diameter of ca. 17 mm; (**H**) Bright field microscope image of as cast film (scale bar represents 100 μm); (**I**) Bright field microscope image of methanol treated film (scale bar represents 100 μm); (**J**) Bright field microscope image of mouse embryo fibroblasts cultured on methanol treated films for 6.5 hours (scale bar represents 100 μm); (**K**) Fluorescence microscope image of Calcein A/M stained fibroblasts cultured on methanol treated films (scale bar represents 100 μm).

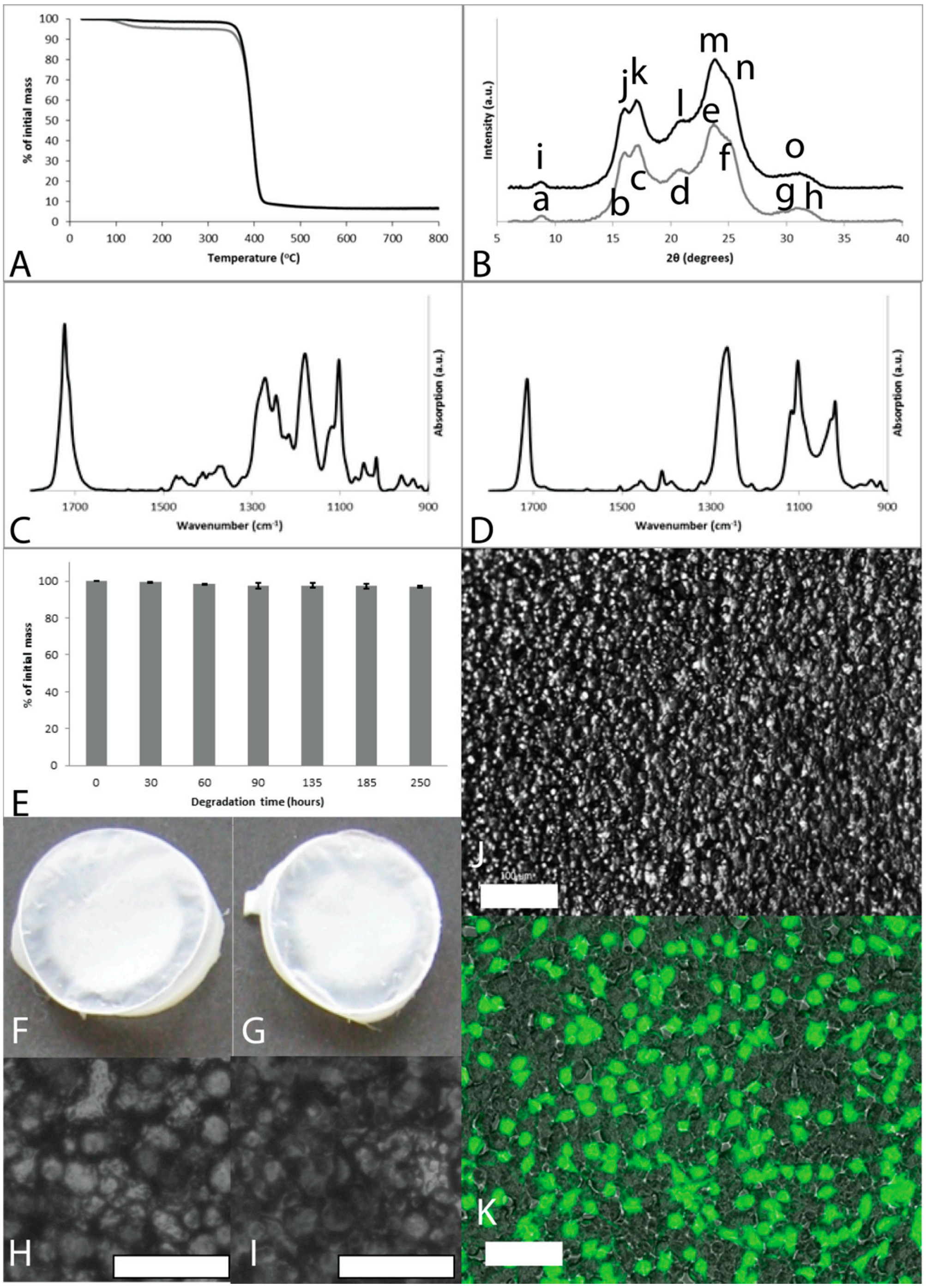

Figure S5. PBT-100 films. (**A**) TGA mass loss profiles, as cast films (grey line) and films after methanol treatment (black line); (**B**) XRD spectra, as cast films (grey line) and films after methanol treatment (black line); lowercase letters label peaks analyzed in Table S1; (**C**) FTIR spectra of as cast films; (**D**) FTIR spectra of films after methanol treatment (right); (**E**) Degradation of methanol treated films upon exposure to a combination of Elastase and Trypsin; (**F**) Photograph of as cast film with diameter of ca. 17 mm; (**G**) Photograph of methanol treated film with diameter of ca. 17 mm; (**H**) Bright field microscope image of as cast film (scale bar represents 100 μm); (**I**) Bright field microscope image of methanol treated film (scale bar represents 100 μm); (**J**) Bright field microscope image of mouse embryo fibroblasts cultured on methanol treated films for 6.5 h (scale bar represents 100 μm); (**K**) Fluorescence microscope image of Calcein A/M stained fibroblasts cultured on methanol treated films (scale bar represents 100 μm).

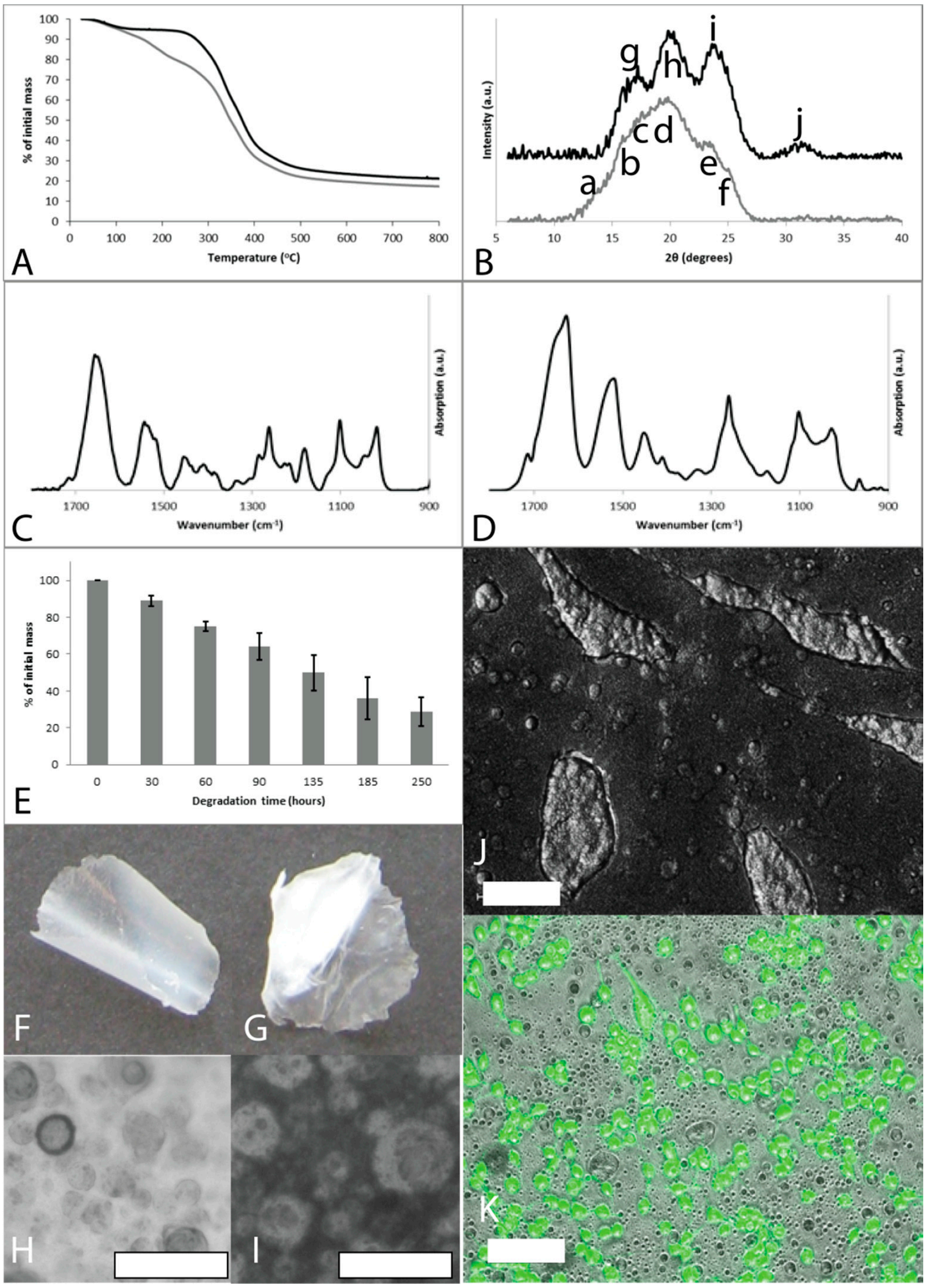

Figure S6. PBTAT-25 films. (**A**) TGA mass loss profiles, as cast films (grey line) and films after methanol treatment (black line); (**B**) XRD spectra, as cast films (grey line) and films after methanol treatment (black line); lowercase letters label peaks analyzed in Table S1; (**C**) FTIR spectra of as cast films; (**D**) FTIR spectra of films after methanol treatment (right); (**E**) Degradation of methanol treated films upon exposure to a combination of Elastase and Trypsin; (**F**) Photograph of as cast film with diameter of ca. 17 mm; (**G**) Photograph of methanol treated film with diameter of ca. 17 mm; (**H**) Bright field microscope image of as cast film (scale bar represents 100 µm); (**I**) Bright field microscope image of methanol treated film (scale bar represents 100 µm); (**J**) Bright field microscope image of mouse embryo fibroblasts cultured on methanol treated films for 6.5 h (scale bar represents 100 µm); (**K**) Fluorescence microscope image of Calcein A/M stained fibroblasts cultured on methanol treated films (scale bar represents 100 µm).

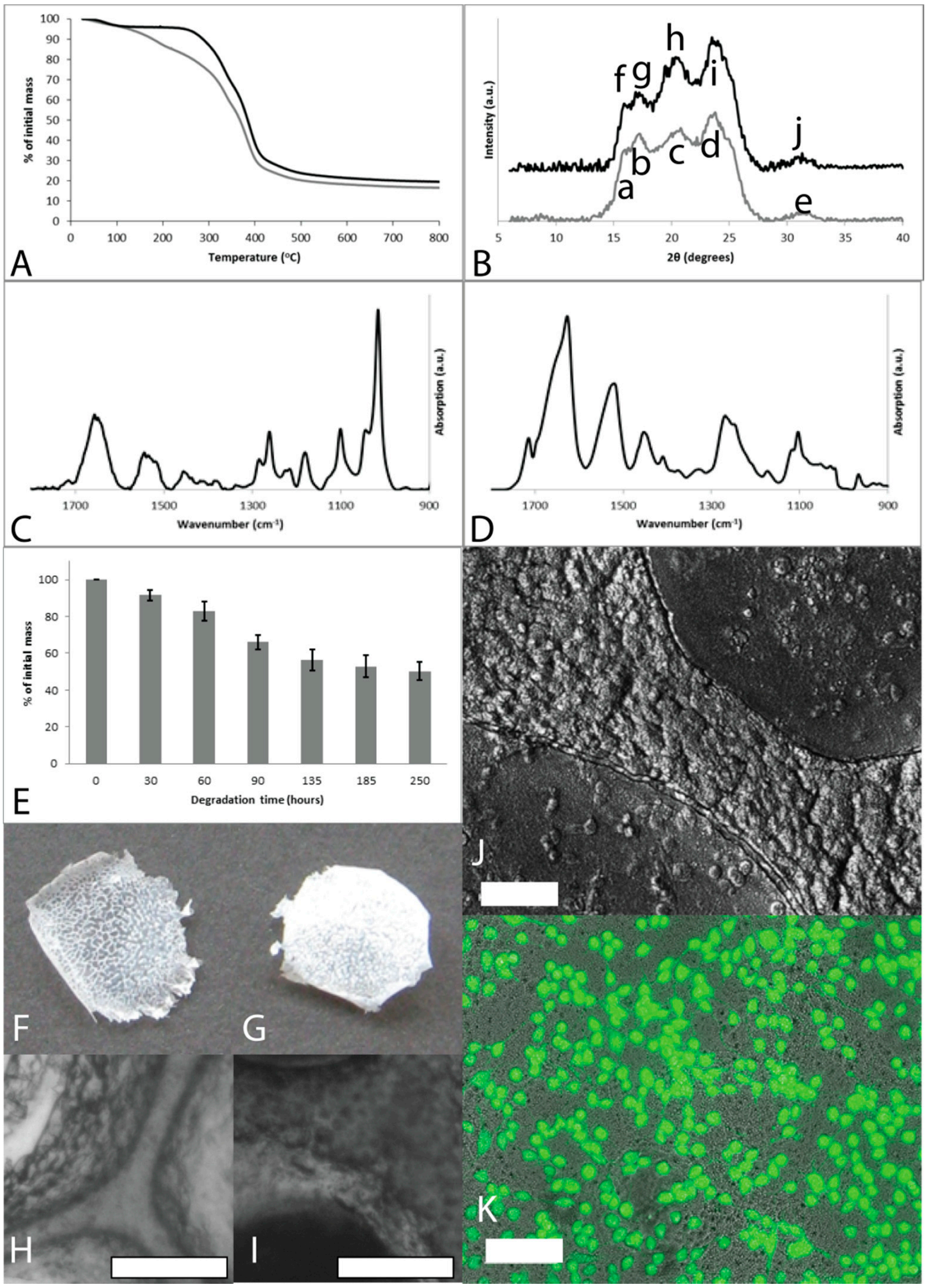

Figure S7. PBTAT-50 films. (**A**) TGA mass loss profiles, as cast films (grey line) and films after methanol treatment (black line); (**B**) XRD spectra, as cast films (grey line) and films after methanol treatment (black line); lowercase letters label peaks analyzed in Table S1; (**C**) FTIR spectra of as cast films; (**D**) FTIR spectra of films after methanol treatment (right); (**E**) Degradation of methanol treated films upon exposure to a combination of Elastase and Trypsin; (**F**) Photograph of as cast film with diameter of ca. 17 mm; (**G**) Photograph of methanol treated film with diameter of ca. 17 mm; (**H**) Bright field microscope image of as cast film (scale bar represents 100 μm); (**I**) Bright field microscope image of methanol treated film (scale bar represents 100 μm); (**J**) Bright field microscope image of mouse embryo fibroblasts cultured on methanol treated films for 6.5 h (scale bar represents 100 μm); (**K**) Fluorescence microscope image of Calcein A/M stained fibroblasts cultured on methanol treated films (scale bar represents 100 μm).

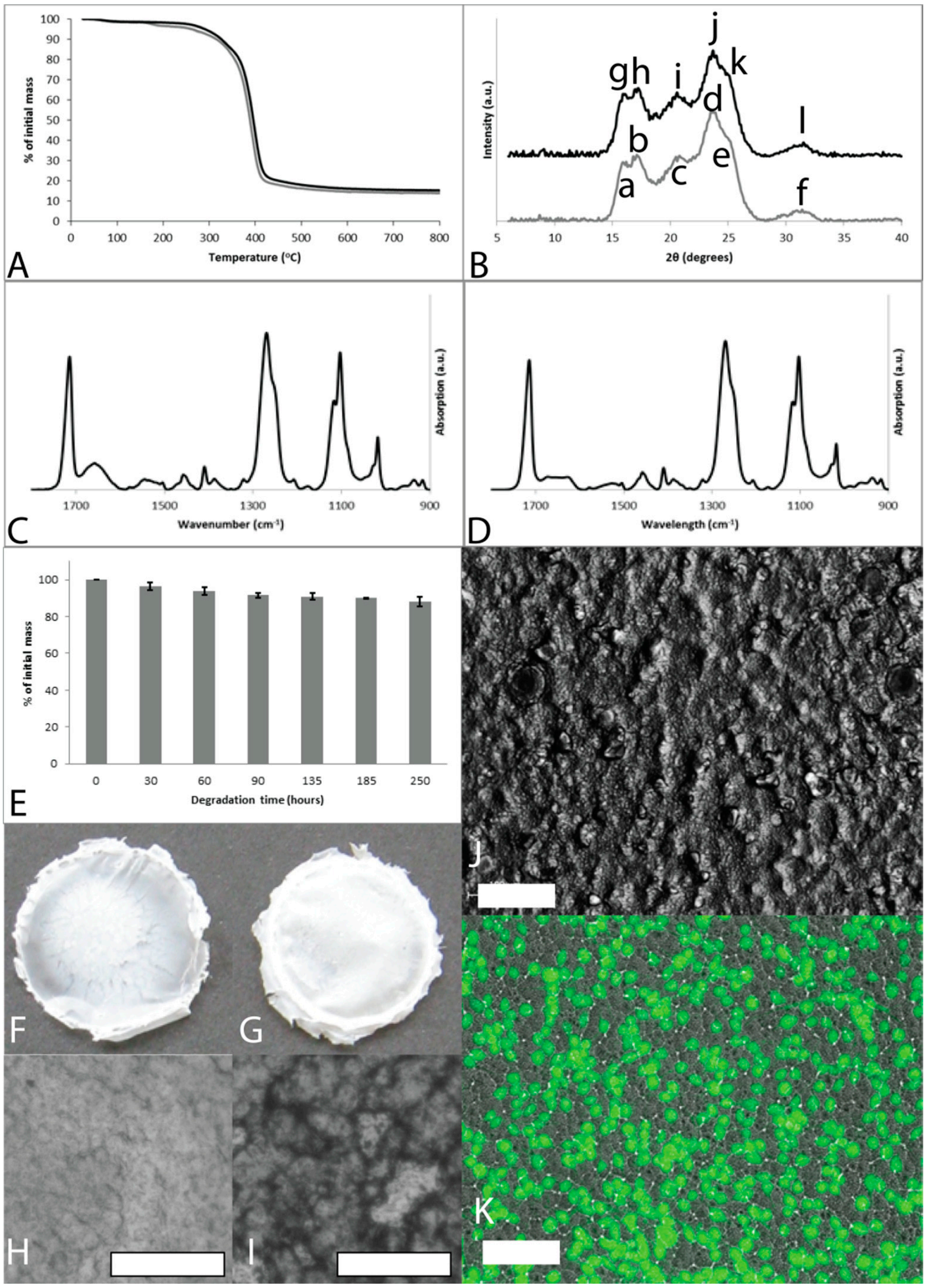

Figure S8. PBTAT-75 films. (**A**) TGA mass loss profiles, as cast films (grey line) and films after methanol treatment (black line); (**B**) XRD spectra, as cast films (grey line) and films after methanol treatment (black line); lowercase letters label peaks analyzed in Table S1; (**C**) FTIR spectra of as cast films; (**D**) FTIR spectra of films after methanol treatment (right); (**E**) Degradation of methanol treated films upon exposure to a combination of Elastase and Trypsin; (**F**) Photograph of as cast film with diameter of ca. 17 mm; (**G**) Photograph of methanol treated film with diameter of ca. 17 mm; (**H**) Bright field microscope image of as cast film (scale bar represents 100 μm); (**I**) Bright field microscope image of methanol treated film (scale bar represents 100 μm); (**J**) Bright field microscope image of mouse embryo fibroblasts cultured on methanol treated films for 6.5 h (scale bar represents 100 μm); (**K**) Fluorescence microscope image of Calcein A/M stained fibroblasts cultured on methanol treated films (scale bar represents 100 μm).

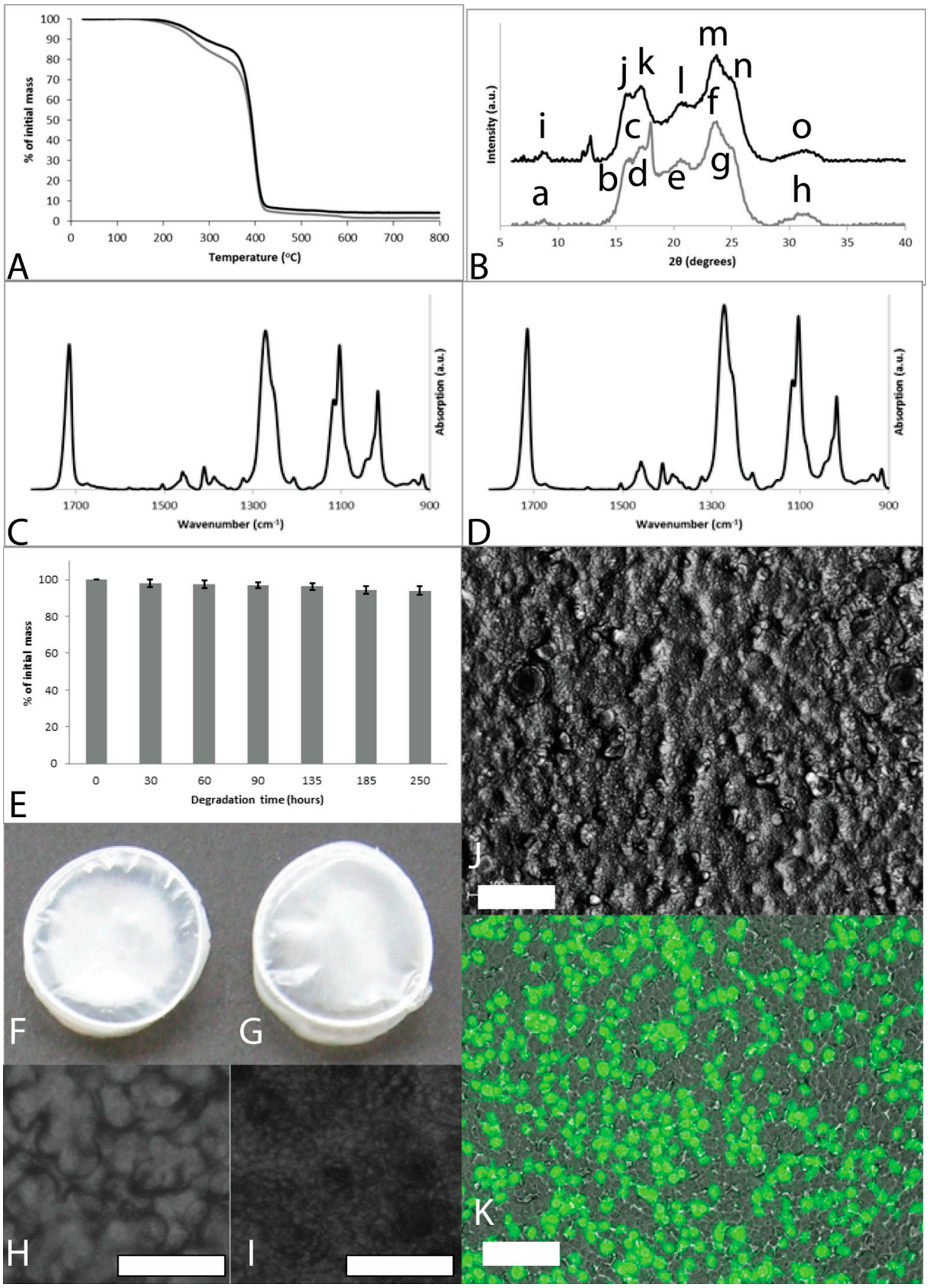

Figure S9. PBTAT-100 films. (**A**) TGA mass loss profiles, as cast films (grey line) and films after methanol treatment (black line); (**B**) XRD spectra, as cast films (grey line) and films after methanol treatment (black line); lowercase letters label peaks analyzed in Table S1; (**C**) FTIR spectra of as cast films; (**D**) FTIR spectra of films after methanol treatment (right); (**E**) Degradation of methanol treated films upon exposure to a combination of Elastase and Trypsin; (**F**) Photograph of as cast film with diameter of ca. 17 mm; (**G**) Photograph of methanol treated film with diameter of ca. 17 mm; (**H**) Bright field microscope image of as cast film (scale bar represents 100 μm); (**I**) Bright field microscope image of methanol treated film (scale bar represents 100 μm); (**J**) Bright field microscope image of mouse embryo fibroblasts cultured on methanol treated films for 6.5 hours (scale bar represents 100 μm); (**K**) Fluorescence microscope image of Calcein A/M stained fibroblasts cultured on methanol treated films (scale bar represents 100 μm).

Table S1. Positions of XRD peaks of films determined using Jade 9 XRD Pattern Processing software.

Film	As Cast			Methanol Treated		
**Sample**	**Peak Label**	**2 θ (Degrees)**	**d-Spacing (Å)**	**Peak Label**	**2 θ (Degrees)**	**d-Spacing (Å)**
eADF-4(C_{16})				Figure S1B, c	16.71	5.30
	Figure S1B, a	14.41	6.14	Figure S1B, d	19.92	4.45
	Figure S1B, b	19.43	4.56	Figure S1B, e	24.01	3.70
				Figure S1B, f	31.86	2.81
PBT-25	Figure S2B, a	15.63	5.66	Figure S2B, f	15.60	5.67
	Figure S2B, b	17.21	5.15	Figure S2B, g	17.16	5.16
	Figure S2B, c	20.02	4.43	Figure S2B, h	19.61	4.52
	Figure S2B, d	21.74	4.09	Figure S2B, i	19.78	4.48
	Figure S2B, e	24.21	3.67	Figure S2B, j	24.11	3.69
				Figure S2B, k	31.20	2.86
PBT-50	Figure S3B, a	15.98	5.54	Figure S3B, f	15.91	5.57
	Figure S3B, b	20.57	4.31	Figure S3B, g	17.46	5.07
	Figure S3B, c	23.62	3.76	Figure S3B, h	20.59	4.31
	Figure S3B, d	24.62	3.61	Figure S3B, i	23.25	3.82
	Figure S3B, e	31.69	2.82	Figure S3B, j	24.46	3.64
				Figure S3B, k	31.69	2.82
PBT-75	Figure S4B, a	15.82	5.59	Figure S4B, g	15.75	5.62
	Figure S4B, b	17.27	5.13	Figure S4B, h	16.84	5.26
	Figure S4B, c	20.37	4.36	Figure S4B, i	20.68	4.29
	Figure S4B, d	22.40	3.97	Figure S4B, j	23.66	4.40
	Figure S4B, e	24.26	3.67	Figure S4B, k	24.70	3.60
	Figure S4B, f	31.88	2.80	Figure S4B, l	31.30	2.86
PBT-100	Figure S5B, a	8.82	10.01	Figure S5B, i	8.92	9.90
	Figure S5B, b	15.92	5.56	Figure S5B, j	15.85	5.58
	Figure S5B, c	17.21	5.15	Figure S5B, k	17.10	5.18
	Figure S5B, d	21.50	4.13	Figure S5B, l	21.10	4.20
	Figure S5B, e	23.76	3.74	Figure S5B, m	23.74	3.74
	Figure S5B, f	25.20	3.53	Figure S5B, n	25.19	3.53
	Figure S5B, g	30.98	2.88	Figure S5B, o	31.30	2.85
	Figure S5B, h	31.79	2.81			
PBTAT-25	Figure S6B, a	14.35	6.16			
	Figure S6B, b	15.82	5.59	Figure S6B, g	16.69	5.30
	Figure S6B, c	17.23	5.14	Figure S6B, h	19.90	4.46
	Figure S6B, d	20.21	4.39	Figure S6B, i	23.98	3.71
	Figure S6B, e	22.94	3.87	Figure S6B, j	31.30	2.86
	Figure S6B, f	24.63	3.61			
PBTAT-50	Figure S7B, a	15.79	5.61	Figure S7B, f	15.83	5.59
	Figure S7B, b	17.11	5.18	Figure S7B, g	16.92	5.24
	Figure S7B, c	20.38	4.35	Figure S7B, h	20.56	4.32
	Figure S7B, d	23.53	3.78	Figure S7B, i	23.43	3.79
	Figure S7B, e	31.43	2.84	Figure S7B, j	31.25	2.86
PBTAT-75	Figure S8B, a	15.92	5.56	Figure S8B, g	15.98	5.54
	Figure S8B, b	17.15	5.1	Figure S8B, h	17.24	5.14
	Figure S8B, c	20.70	4.29	Figure S8B, i	20.67	4.29
	Figure S8B, d	23.51	3.78	Figure S8B, j	23.41	3.79
	Figure S8B, e	24.76	3.59	Figure S8B, k	24.66	3.61
	Figure S8B, f	31.48	2.84	Figure S8B, l	31.33	2.85
PBTAT-100	Figure S9B, a	8.89	9.94	Figure S9B, i	8.89	9.94
	Figure S9B, b	16.03	5.52	Figure S9B, j	15.81	5.60
	Figure S9B, c	17.27	5.13	Figure S9B, k	17.09	5.18
	Figure S9B, d	17.96	4.93	Figure S9B, l	20.61	4.31
	Figure S9B, e	20.93	4.24	Figure S9B, m	23.52	3.78
	Figure S9B, f	23.44	3.79	Figure S9B, n	25.08	3.55
	Figure S9B, g	24.89	3.57	Figure S9B, o	31.43	2.84
	Figure S9B, h	31.05	2.88			

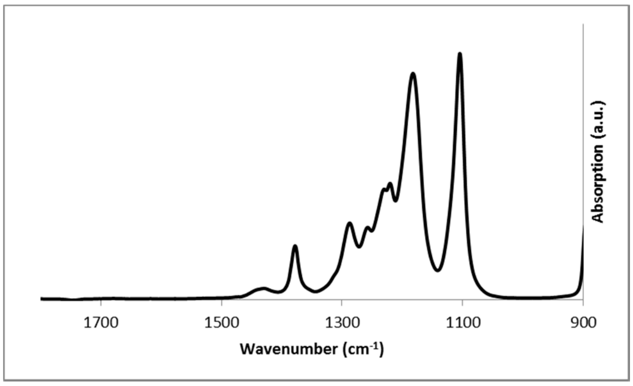

Figure S10. FTIR spectrum of pure HFIP.

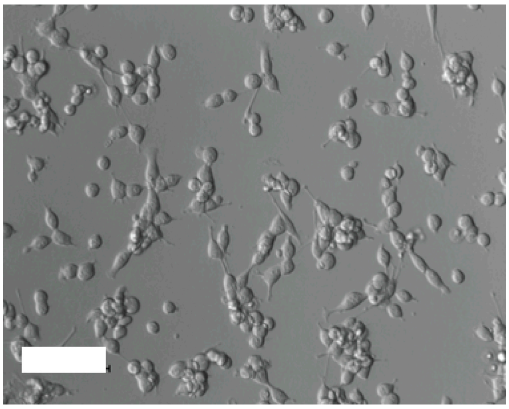

Figure S11. Bright field microscope image of fibroblasts cultured on Nunclon® Tissue Culture Plate (scale bar represents 100 μm).
